# Supplementary material for: Modeling glioblastoma heterogeneity as a dynamic network of cell states
Source: Mol Syst Biol. 2021 Sep 16;17(9):e10105. doi: 10.15252/msb.202010105 (PMC8444284; doi:10.15252/msb.202010105)
Supplement: Supplementary file 6 — Source Data for Figure 5 [file MSB-17-e10105-s004.zip › Figure5A_sourcedata/GSEA_3071/nosigdb_stateIII.GseaPreranked.1622795155562/RICHARDS_NOWAKOWSKI_SCIENCE_2017_NIN2_UPREG.html]

Details for gene set RICHARDS\_NOWAKOWSKI\_SCIENCE\_2017\_NIN2\_UPREG[GSEA]

|  || Dataset | state33071 |
| Phenotype | NoPhenotypeAvailable |
| Upregulated in class | na\_pos |
| GeneSet | RICHARDS\_NOWAKOWSKI\_SCIENCE\_2017\_NIN2\_UPREG |
| Enrichment Score (ES) | 0.35638434 |
| Normalized Enrichment Score (NES) | 5.694819 |
| Nominal p-value | 0.0 |
| FDR q-value | 0.0 |
| FWER p-Value | 0.0 |
Table: GSEA Results Summary

  

Fig 1: Enrichment plot: RICHARDS\_NOWAKOWSKI\_SCIENCE\_2017\_NIN2\_UPREG      
 Profile of the Running ES Score & Positions of GeneSet Members on the Rank Ordered List

  

| PROBE | GENE SYMBOL | GENE\_TITLE | RANK IN GENE LIST | RANK METRIC SCORE | RUNNING ES | CORE ENRICHMENT || 1 | RPL38 |  |  | 5 | 0.638 | 0.0077 | Yes |
| 2 | RPS29 |  |  | 7 | 0.630 | 0.0163 | Yes |
| 3 | RPL27 |  |  | 11 | 0.587 | 0.0237 | Yes |
| 4 | RPL6 |  |  | 16 | 0.555 | 0.0305 | Yes |
| 5 | RPL35 |  |  | 17 | 0.549 | 0.0382 | Yes |
| 6 | RPS25 |  |  | 19 | 0.527 | 0.0454 | Yes |
| 7 | RPL24 |  |  | 21 | 0.524 | 0.0525 | Yes |
| 8 | RPL22 |  |  | 22 | 0.520 | 0.0598 | Yes |
| 9 | SH3BGRL3 |  |  | 26 | 0.505 | 0.0661 | Yes |
| 10 | UBA52 |  |  | 33 | 0.494 | 0.0715 | Yes |
| 11 | RPS13 |  |  | 35 | 0.490 | 0.0781 | Yes |
| 12 | RPLP2 |  |  | 36 | 0.489 | 0.0850 | Yes |
| 13 | RPS21 |  |  | 39 | 0.482 | 0.0913 | Yes |
| 14 | TXN |  |  | 47 | 0.465 | 0.0960 | Yes |
| 15 | RPS20 |  |  | 49 | 0.463 | 0.1022 | Yes |
| 16 | RPL18A |  |  | 58 | 0.453 | 0.1065 | Yes |
| 17 | RPL36 |  |  | 62 | 0.449 | 0.1120 | Yes |
| 18 | RPL37A |  |  | 64 | 0.448 | 0.1180 | Yes |
| 19 | ZFAS1 |  |  | 65 | 0.447 | 0.1243 | Yes |
| 20 | RPL26 |  |  | 66 | 0.447 | 0.1306 | Yes |
| 21 | FTL |  |  | 67 | 0.445 | 0.1369 | Yes |
| 22 | RPL39 |  |  | 72 | 0.442 | 0.1421 | Yes |
| 23 | RPS28 |  |  | 73 | 0.440 | 0.1483 | Yes |
| 24 | RPL11 |  |  | 76 | 0.436 | 0.1539 | Yes |
| 25 | RPS3A |  |  | 82 | 0.429 | 0.1586 | Yes |
| 26 | RPS19 |  |  | 84 | 0.427 | 0.1643 | Yes |
| 27 | RPS27A |  |  | 95 | 0.413 | 0.1675 | Yes |
| 28 | RPL35A |  |  | 96 | 0.413 | 0.1733 | Yes |
| 29 | PFDN1 |  |  | 97 | 0.413 | 0.1791 | Yes |
| 30 | RPL34 |  |  | 105 | 0.407 | 0.1830 | Yes |
| 31 | FAU |  |  | 113 | 0.400 | 0.1868 | Yes |
| 32 | TBCA |  |  | 114 | 0.400 | 0.1924 | Yes |
| 33 | UQCRB |  |  | 117 | 0.398 | 0.1975 | Yes |
| 34 | RPS27 |  |  | 132 | 0.388 | 0.1992 | Yes |
| 35 | COX6B1 |  |  | 134 | 0.387 | 0.2044 | Yes |
| 36 | DNAJC8 |  |  | 136 | 0.384 | 0.2096 | Yes |
| 37 | SNHG5 |  |  | 138 | 0.383 | 0.2147 | Yes |
| 38 | NDUFA4 |  |  | 140 | 0.382 | 0.2198 | Yes |
| 39 | RPL21 |  |  | 141 | 0.382 | 0.2252 | Yes |
| 40 | NDUFA1 |  |  | 143 | 0.381 | 0.2303 | Yes |
| 41 | TMSB10 |  |  | 148 | 0.376 | 0.2345 | Yes |
| 42 | SNRPF |  |  | 160 | 0.367 | 0.2368 | Yes |
| 43 | CACYBP |  |  | 165 | 0.364 | 0.2408 | Yes |
| 44 | NEDD8 |  |  | 168 | 0.362 | 0.2454 | Yes |
| 45 | COX7C |  |  | 170 | 0.362 | 0.2502 | Yes |
| 46 | COMMD6 |  |  | 173 | 0.359 | 0.2548 | Yes |
| 47 | CBX1 |  |  | 177 | 0.357 | 0.2590 | Yes |
| 48 | RPL18 |  |  | 178 | 0.355 | 0.2640 | Yes |
| 49 | UBL5 |  |  | 182 | 0.352 | 0.2682 | Yes |
| 50 | RPS27L |  |  | 188 | 0.348 | 0.2717 | Yes |
| 51 | PSME1 |  |  | 190 | 0.347 | 0.2764 | Yes |
| 52 | C19orf53 |  |  | 197 | 0.340 | 0.2796 | Yes |
| 53 | POLR2J |  |  | 215 | 0.331 | 0.2797 | Yes |
| 54 | PTMA |  |  | 222 | 0.327 | 0.2827 | Yes |
| 55 | MZT2B |  |  | 244 | 0.313 | 0.2815 | Yes |
| 56 | RPS15A |  |  | 245 | 0.312 | 0.2859 | Yes |
| 57 | RPL7A |  |  | 255 | 0.308 | 0.2879 | Yes |
| 58 | RPS16 |  |  | 256 | 0.308 | 0.2922 | Yes |
| 59 | TOMM7 |  |  | 264 | 0.303 | 0.2946 | Yes |
| 60 | SEPT7 |  |  | 267 | 0.303 | 0.2984 | Yes |
| 61 | RPL9 |  |  | 269 | 0.302 | 0.3024 | Yes |
| 62 | RPS4Y1 |  |  | 272 | 0.301 | 0.3061 | Yes |
| 63 | TMSB4X |  |  | 273 | 0.300 | 0.3103 | Yes |
| 64 | NDUFS5 |  |  | 275 | 0.299 | 0.3142 | Yes |
| 65 | ARF1 |  |  | 281 | 0.296 | 0.3171 | Yes |
| 66 | RPL10 |  |  | 285 | 0.296 | 0.3204 | Yes |
| 67 | STMN1 |  |  | 298 | 0.291 | 0.3213 | Yes |
| 68 | HMGN1 |  |  | 300 | 0.290 | 0.3252 | Yes |
| 69 | NDUFB3 |  |  | 312 | 0.288 | 0.3263 | Yes |
| 70 | MINOS1 |  |  | 321 | 0.286 | 0.3282 | Yes |
| 71 | TIMM8B |  |  | 322 | 0.286 | 0.3322 | Yes |
| 72 | AIMP1 |  |  | 336 | 0.282 | 0.3328 | Yes |
| 73 | MRPS5 |  |  | 342 | 0.280 | 0.3354 | Yes |
| 74 | NDUFA5 |  |  | 346 | 0.278 | 0.3385 | Yes |
| 75 | RPS11 |  |  | 358 | 0.272 | 0.3394 | Yes |
| 76 | C11orf58 |  |  | 365 | 0.271 | 0.3416 | Yes |
| 77 | TOP1 |  |  | 382 | 0.267 | 0.3411 | Yes |
| 78 | FKBP3 |  |  | 387 | 0.264 | 0.3438 | Yes |
| 79 | CCDC90B |  |  | 390 | 0.263 | 0.3470 | Yes |
| 80 | RPL7 |  |  | 394 | 0.262 | 0.3498 | Yes |
| 81 | ZCRB1 |  |  | 395 | 0.261 | 0.3535 | Yes |
| 82 | TPRKB |  |  | 399 | 0.260 | 0.3564 | Yes |
| 83 | HNRNPH1 |  |  | 426 | 0.252 | 0.3530 | No |
| 84 | NDUFB8 |  |  | 428 | 0.251 | 0.3563 | No |
| 85 | RALGAPA1 |  |  | 480 | -0.253 | 0.3463 | No |
| 86 | SBF2 |  |  | 508 | -0.255 | 0.3427 | No |
| 87 | ICA1L |  |  | 526 | -0.255 | 0.3418 | No |
| 88 | PTPRA |  |  | 552 | -0.257 | 0.3388 | No |
| 89 | KMT2A |  |  | 773 | -0.268 | 0.2840 | No |
| 90 | CDK13 |  |  | 779 | -0.268 | 0.2865 | No |
| 91 | MAF |  |  | 800 | -0.269 | 0.2850 | No |
| 92 | MSI2 |  |  | 848 | -0.272 | 0.2763 | No |
| 93 | FEZ1 |  |  | 1005 | -0.280 | 0.2387 | No |
| 94 | NBPF12 |  |  | 1036 | -0.281 | 0.2347 | No |
| 95 | CD46 |  |  | 1039 | -0.281 | 0.2382 | No |
| 96 | ZNF250 |  |  | 1152 | -0.288 | 0.2124 | No |
| 97 | VPS13B |  |  | 1183 | -0.289 | 0.2085 | No |
| 98 | TIAM1 |  |  | 1276 | -0.294 | 0.1882 | No |
| 99 | SLC35E3 |  |  | 1444 | -0.303 | 0.1481 | No |
| 100 | GRIA4 |  |  | 1488 | -0.305 | 0.1409 | No |
| 101 | KIDINS220 |  |  | 1589 | -0.311 | 0.1187 | No |
| 102 | DNM3 |  |  | 1676 | -0.317 | 0.1003 | No |
| 103 | EAF1 |  |  | 1746 | -0.321 | 0.0865 | No |
| 104 | BMPR2 |  |  | 1779 | -0.323 | 0.0826 | No |
| 105 | ZNF91 |  |  | 1803 | -0.325 | 0.0810 | No |
| 106 | GPATCH2L |  |  | 1882 | -0.329 | 0.0649 | No |
| 107 | HIP1 |  |  | 1905 | -0.330 | 0.0637 | No |
| 108 | LBR |  |  | 1973 | -0.334 | 0.0506 | No |
| 109 | PKD2 |  |  | 1977 | -0.334 | 0.0545 | No |
| 110 | PCDH17 |  |  | 2043 | -0.338 | 0.0420 | No |
| 111 | MTDH |  |  | 2083 | -0.340 | 0.0364 | No |
| 112 | MARK1 |  |  | 2087 | -0.341 | 0.0404 | No |
| 113 | DENND4C |  |  | 2157 | -0.346 | 0.0269 | No |
| 114 | ZNF704 |  |  | 2210 | -0.350 | 0.0180 | No |
| 115 | PPM1L |  |  | 2234 | -0.351 | 0.0169 | No |
| 116 | MAGI1 |  |  | 2340 | -0.361 | -0.0060 | No |
| 117 | ARX |  |  | 2382 | -0.364 | -0.0118 | No |
| 118 | GNAQ |  |  | 2392 | -0.364 | -0.0090 | No |
| 119 | PDZRN4 |  |  | 2461 | -0.371 | -0.0219 | No |
| 120 | ZEB2 |  |  | 2484 | -0.372 | -0.0225 | No |
| 121 | ABAT |  |  | 2521 | -0.375 | -0.0268 | No |
| 122 | SOX4 |  |  | 2595 | -0.381 | -0.0408 | No |
| 123 | NRDE2 |  |  | 2603 | -0.382 | -0.0373 | No |
| 124 | TACC2 |  |  | 2615 | -0.383 | -0.0348 | No |
| 125 | FAM214A |  |  | 2679 | -0.389 | -0.0461 | No |
| 126 | MIDN |  |  | 2691 | -0.390 | -0.0435 | No |
| 127 | SCARB2 |  |  | 2697 | -0.390 | -0.0394 | No |
| 128 | CAMK4 |  |  | 2734 | -0.394 | -0.0434 | No |
| 129 | KIF21A |  |  | 2786 | -0.398 | -0.0514 | No |
| 130 | RPS6KA5 |  |  | 2789 | -0.398 | -0.0463 | No |
| 131 | MAGEH1 |  |  | 2889 | -0.407 | -0.0669 | No |
| 132 | ZBTB21 |  |  | 2952 | -0.414 | -0.0775 | No |
| 133 | DCLK2 |  |  | 3039 | -0.425 | -0.0944 | No |
| 134 | NOVA2 |  |  | 3061 | -0.427 | -0.0940 | No |
| 135 | ARIH1 |  |  | 3098 | -0.431 | -0.0975 | No |
| 136 | AKAP9 |  |  | 3154 | -0.438 | -0.1059 | No |
| 137 | PDZRN3 |  |  | 3176 | -0.441 | -0.1053 | No |
| 138 | ADAMTS5 |  |  | 3188 | -0.441 | -0.1020 | No |
| 139 | SOX11 |  |  | 3205 | -0.443 | -0.1000 | No |
| 140 | GABBR1 |  |  | 3226 | -0.447 | -0.0991 | No |
| 141 | KLHL28 |  |  | 3280 | -0.456 | -0.1067 | No |
| 142 | NRXN3 |  |  | 3318 | -0.464 | -0.1100 | No |
| 143 | RFX3 |  |  | 3395 | -0.480 | -0.1235 | No |
| 144 | RNF38 |  |  | 3454 | -0.494 | -0.1319 | No |
| 145 | DPYSL3 |  |  | 3498 | -0.507 | -0.1362 | No |
| 146 | CCDC88A |  |  | 3549 | -0.526 | -0.1421 | No |
| 147 | SP9 |  |  | 3553 | -0.528 | -0.1355 | No |
| 148 | SOX2 |  |  | 3613 | -0.555 | -0.1433 | No |
| 149 | LSAMP |  |  | 3623 | -0.561 | -0.1378 | No |
| 150 | GAD1 |  |  | 3644 | -0.574 | -0.1351 | No |
| 151 | DLX1 |  |  | 3665 | -0.589 | -0.1321 | No |
| 152 | SOX6 |  |  | 3675 | -0.596 | -0.1261 | No |
| 153 | KAT6B |  |  | 3684 | -0.602 | -0.1197 | No |
| 154 | TCF4 |  |  | 3696 | -0.612 | -0.1141 | No |
| 155 | KLHL24 |  |  | 3697 | -0.613 | -0.1054 | No |
| 156 | SLAIN1 |  |  | 3718 | -0.631 | -0.1019 | No |
| 157 | DLX6 |  |  | 3719 | -0.632 | -0.0930 | No |
| 158 | CADPS |  |  | 3731 | -0.641 | -0.0869 | No |
| 159 | DLX2 |  |  | 3742 | -0.662 | -0.0802 | No |
| 160 | SYT11 |  |  | 3748 | -0.669 | -0.0721 | No |
| 161 | FZD3 |  |  | 3765 | -0.695 | -0.0666 | No |
| 162 | BCL11B |  |  | 3799 | -0.740 | -0.0649 | No |
| 163 | NCAN |  |  | 3813 | -0.761 | -0.0576 | No |
| 164 | BCL11A |  |  | 3817 | -0.766 | -0.0476 | No |
| 165 | TCEAL7 |  |  | 3834 | -0.798 | -0.0406 | No |
| 166 | DLX5 |  |  | 3855 | -0.850 | -0.0340 | No |
| 167 | CADM1 |  |  | 3856 | -0.851 | -0.0220 | No |
| 168 | DCX |  |  | 3911 | -1.320 | -0.0178 | No |
| 169 | MALAT1 |  |  | 3924 | -1.583 | 0.0013 | No |
Table: GSEA details [plain text format]

  

Fig 2: RICHARDS\_NOWAKOWSKI\_SCIENCE\_2017\_NIN2\_UPREG: Random ES distribution      
 Gene set null distribution of ES for **RICHARDS\_NOWAKOWSKI\_SCIENCE\_2017\_NIN2\_UPREG**
